# COVID-19 salivary Raman fingerprint: innovative approach for the detection of current and past SARS-CoV-2 infections

**DOI:** 10.1038/s41598-021-84565-3

**Published:** 2021-03-02

**Authors:** C. Carlomagno, D. Bertazioli, A. Gualerzi, S. Picciolini, P. I. Banfi, A. Lax, E. Messina, J. Navarro, L. Bianchi, A. Caronni, F. Marenco, S. Monteleone, C. Arienti, M. Bedoni

**Affiliations:** 1 IRCCS Fondazione Don Carlo Gnocchi ONLUS, Via Capecelatro 66, 20148 Milan, Italy; 2 Università di Milano-Bicocca, Viale Sarca 366, 20126 Milan, Italy

**Keywords:** Computational biology and bioinformatics, Data processing, Diagnostic markers, Predictive markers

## Abstract

The pandemic of COVID-19 is continuously spreading, becoming a worldwide emergency. Early and fast identification of subjects with a current or past infection must be achieved to slow down the epidemiological widening. Here we report a Raman-based approach for the analysis of saliva, able to significantly discriminate the signal of patients with a current infection by COVID-19 from healthy subjects and/or subjects with a past infection. Our results demonstrated the differences in saliva biochemical composition of the three experimental groups, with modifications grouped in specific attributable spectral regions. The Raman-based classification model was able to discriminate the signal collected from COVID-19 patients with accuracy, precision, sensitivity and specificity of more than 95%. In order to translate this discrimination from the signal-level to the patient-level, we developed a Deep Learning model obtaining accuracy in the range 89–92%. These findings have implications for the creation of a potential Raman-based diagnostic tool, using saliva as minimal invasive and highly informative biofluid, demonstrating the efficacy of the classification model.

## Introduction

In December 2019, a novel coronavirus named Severe Acute Respiratory Syndrome Coronavirus 2 (SARS-CoV-2) has emerged in Hubei Province of China and has rapidly spread around the globe. The disease caused by the virus was named Coronavirus Disease 2019 (COVID-19) and has become a public health emergency of international concern as declared by the World Health Organization (WHO) ^1^. Nasopharyngeal and oropharyngeal swabs are the recommended specimens for diagnostic tests, with Real Time reverse transcription Polymerase Chain Reaction (rRT-PCR) on respiratory specimens representing the gold standard method for detection of SARS-CoV-2 infection. However, the collection procedure is relatively invasive, it causes discomfort, it can occasionally induce coughing and bleeding and it requires close contact between healthcare workers and patients, which poses a risk of transmission of the virus to nurses and physicians also with the full personal protective equipment. Moreover, the analytical protocol required for the SARS-CoV-2 detection through rRT-PCR requires time, specialized laboratories, expensive reagents and adequate personnel in a process highly exposed to the operator’s error, which can influence the final sensitivity of the assay. Researchers are focalized in studies regarding new technologies for the virus detection in other more accessible and informative biofluids^2^. Hence, this nasopharyngeal and oropharyngeal swabs are not desirable for serial monitoring of viral load and for massive screening. Considering that the primary route of diffusion for SARS-CoV-2 are respiratory droplets and aerosols, saliva was proposed as a valid alternative to nasopharyngeal swabs^3,4^. Saliva is an easily accessible biofluid that can be collected without invasive procedures and by non-specialized personnel. It is an aqueous mixture of molecular components secreted by major (90%) and minor (10%) salivary glands, but it may contain also desquamated epithelial cells, respiratory secretions, molecules circulating in the blood vessels, gastric acids and hormones together with microorganisms and live viruses. Salivary samples were demonstrated to have a high concordance rate with nasopharyngeal specimens in the detection of viruses, including coronaviruses^5^. Moreover, salivary antigen tests have been already proposed as discriminatory COVID-19 assays, but their sensibilities are still incomparable with the one presented by rRT-PCR^6^. In fact, considering patients affected by severe COVID-19, saliva was demonstrated to be a reliable tool for qualitative COVID-19 diagnosis through the rRT-PCR procedure^3,7^. Looking at the temporal profile of SARS-CoV-2 load in saliva, it was found that saliva reached a peak during the first week from symptom onset and then declined^8^. Although the use of saliva can significantly reduce the time and the costs of specimen collection, RT-PCR for the detection of COVID-19 is a time-consuming procedure because sample preparation is required prior to analysis and, despite being highly sensitive, it has a high false negative rate^9^. To overcome the rRT-PCR methodological limitation, easy to perform and reliable immunoassays have been developed, but their limit relies on their target, i.e. IgA, IgM and IgG antibodies directed against the virus that are reported to increase in most patients one week after the infection both in blood and saliva, with characteristic expression patterns up to 3 months after the infection^8,10,11^. The use of saliva represents a good alternative for antibody testing for SARS-CoV-2 by immunoassays, showing a direct correlation between the serum and saliva data^10,12,13^. The immunological method circumvents the extraction of virus nucleic acid and shortens the detection time; however, it is not applicable for the early diagnosis of COVID-19 due to the low antibody concentration at the beginning. In this framework, vibrational spectroscopies like Raman spectroscopy (RS) are promising alternatives in molecular diagnostics. These techniques have already proved their applicability in the diagnosis of infections at the point-of-care, with promising results also for early diagnosis and monitoring of multiple human diseases^14^. Besides the application in cell and tissue analysis for cancer detection, RS has demonstrated its reliability and promising applicability also in the analysis of complex biological samples like saliva for disease detection and monitoring^15^. The use of saliva as substrate for Raman analysis has demonstrated unparalleled advantages related mainly to the limited sample processing required, the negligible invasiveness of sample collection, coupled to the multitude of molecules and disease biomarkers that can be investigated for clinical purposes^16^. Taking advantage of Surface Enhanced Raman Scattering (SERS), RS challenges current fluorescent based detection methods in terms of both sensitivity and multiplexing ability, detecting multiple components in a mixture, including viruses, in a point-of-care platforms^17–19^. SERS has been proposed for the study of SARS-CoV-2, with nanoparticle-based biosensors used to detect SARS-CoV-2 spike proteins^20^, still the remarkable advantages of vibrational spectroscopy have not been proposed for clinical application in COVID-19 pandemic. A potential help in the managing and decodification of the huge amount of data provided by RS, can be found in the application of Machine Learning (ML) and Deep Learning (DL) algorithms^21^. The computational approach for the creation of classification models in the biological and spectroscopic fields has been already proposed and applied for different applications including diagnostics, metabolomics, proteomics and genomics^22–25^. The application of ML/DL methods provides a powerful instrument for the data analysis, providing information about hidden trends, connections and correlations. In the present study, we propose the use of SERS to identify a Raman fingerprint of SARS-CoV-2 in the saliva of patients affected by COVID-19. Thanks to the application of classification models based on ML and DL techniques, we created a fast tool for the discrimination of the COVID-19 condition. Interestingly, our data provide evidence that the saliva of patients with current infection by SARS-CoV-2 presents a biochemical signature that allows their fast detection and discrimination from people with a past SARS-CoV-2 infection and from healthy subjects, with accuracy, precision, specificity and sensitivity of more than 90%. The correlation with the extracted data confirmed the reliability of the method, demonstrating statistical correlation with the clinical scales used for the COVID-19 severity classification and with the time between the first positive SARS-CoV-2 test and the last negative.

## Results

### COVID-19 Raman fingerprint

**Figure 1:**
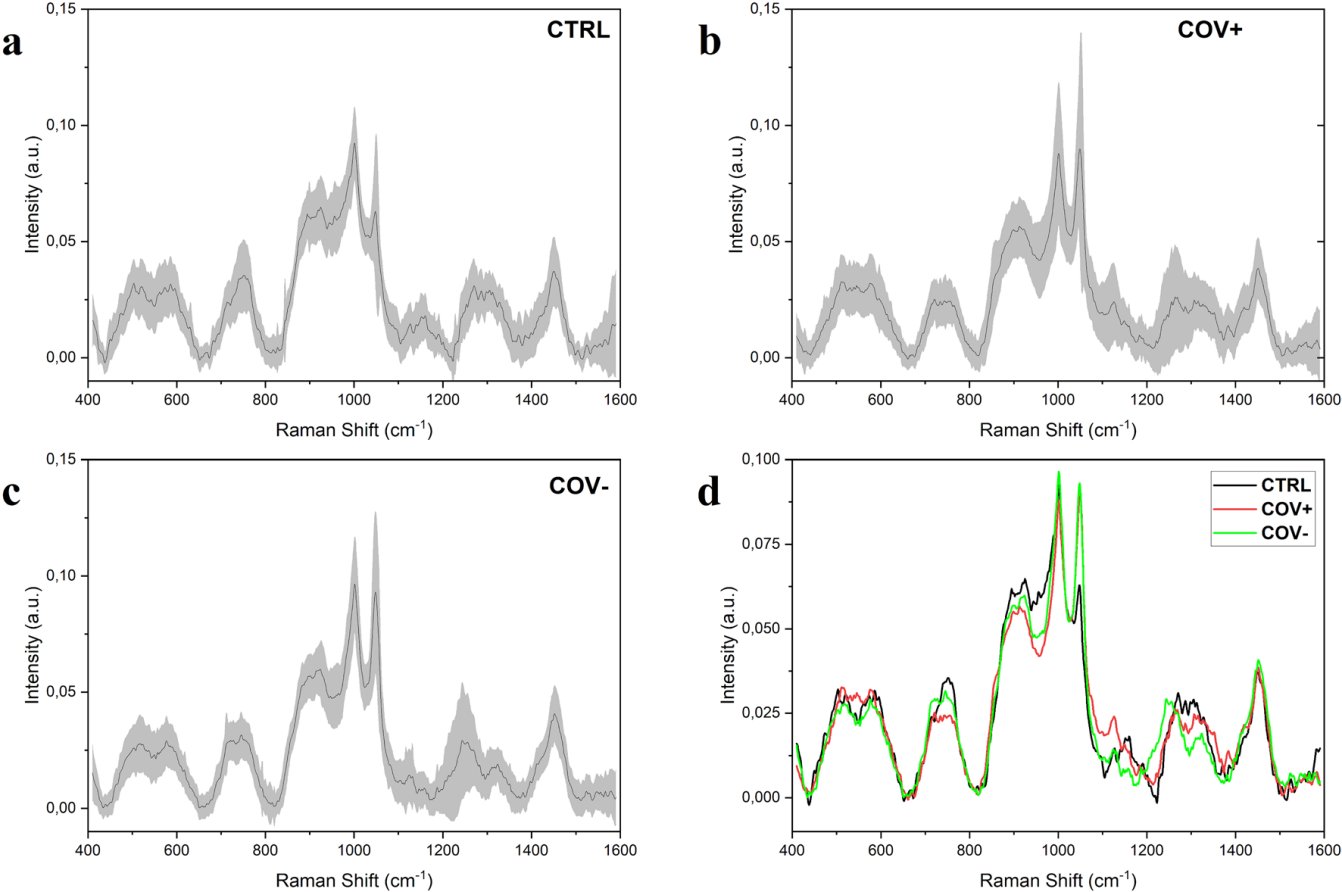
Average Raman spectra obtained from **(a)** healthy subjects (CTRL), **(b)** patients affected by COVID-19 (COV +) and **(c)** subjects with at least two negative SARS-CoV-2 tests after being positive (COV−). **(d)** Overlapped average spectra of the three experimental groups highlighting spectral differences. The grey bands represent the standard deviations.

**Figure 2:**
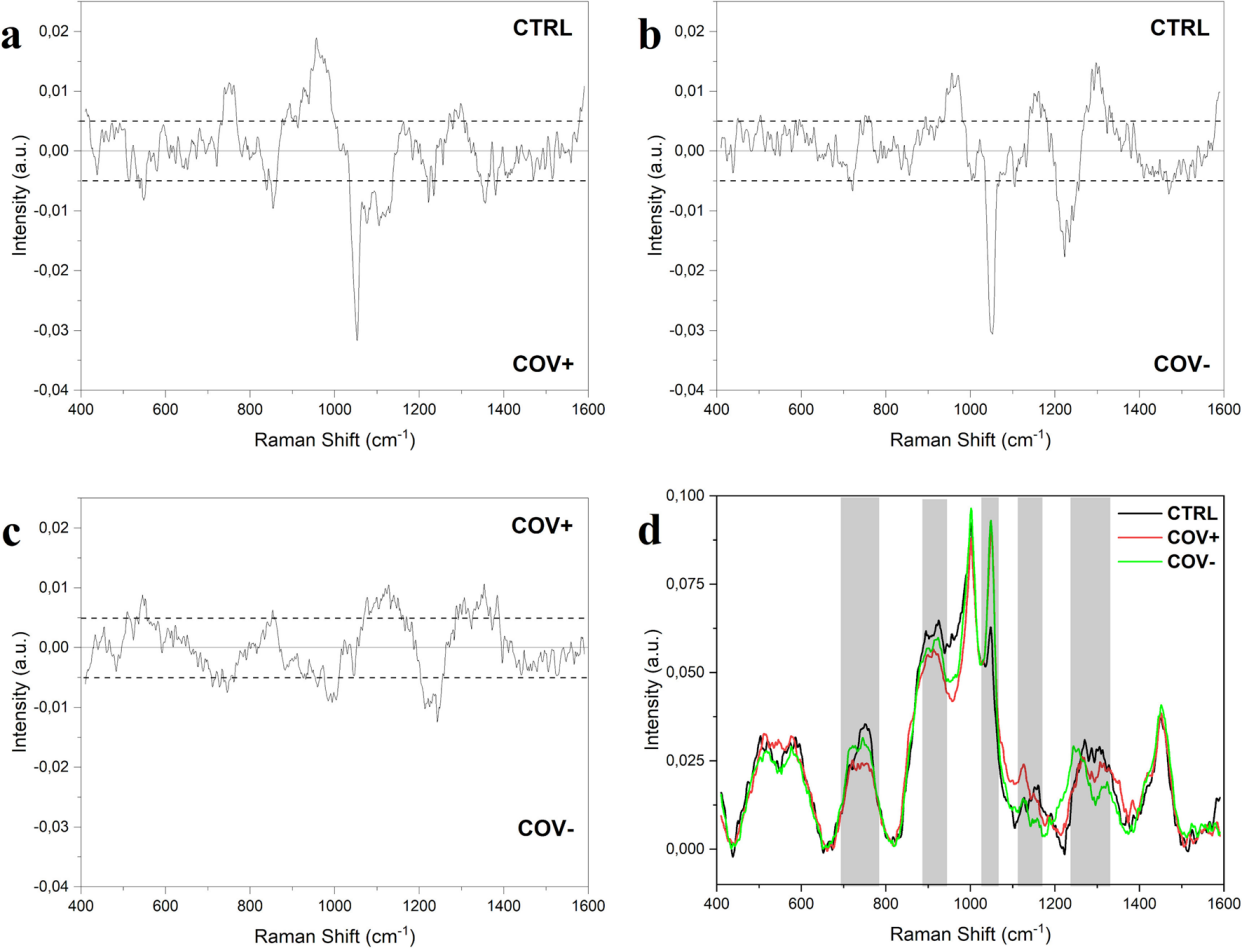
Subtraction Raman spectra of (**a)** the average CTRL signal versus the average signal of COV+  , **(b)** the average CTRL signal versus the average COV- spectrum and c) the average COV+  signal versus the average COV− signal. The ± 0.005 ∆I interval is indicated in the graphs, confirmed by the error propagation from the spectra standard deviation. d) Overlapped average spectra of CTRL, COV+  and COV− with the main different regions highlighted.

The salivary Raman analysis was carried out following and slightly modifying the analytical protocol developed and validated by our group, leading to the observation of the whole biochemical pattern of saliva^15^. The sample preparation procedure was reduced avoiding the filtration step, making in this way the analysis faster, cheaper and more informative, modifying consecutively the Raman acquisition parameters. The procedure was intended for the safe collection of saliva, limiting the sample handling and difficult or complex passages. Figure 1 shows the salivary spectra obtained from the considered experimental groups, Healthy Subjects (CTRL) Fig. 1a, patients affected by COVID-19 (COV +) Fig. 1b, and subjects negative to the SARS-CoV-2 test with an ascertained episode of COVID-19 (COV−) Fig. 1c. The principal characteristics peaks and bands are located at 509, 577, 716, 748, 897, 922, 1000, 1048, 1126, 1155, 1249, 1288, 1317, 1384 and 1453 cm^−1^ highlighted in the overlapped spectrum (Fig. 1d). The presented spectra are mainly dominated by the peaks attributed to C–N stretching and CH_3_ rocking in protein backbone (897 and 1155 cm^−1^) and by the signal at 1453 cm^−1^ assigned to the C–H stretching of glycoproteins, mostly generated from mucines ^26,27^. Crucial importance in the signal discrimination between the different experimental groups can be attributed to the peaks located at 748, 922, 1048, 1249 and 1126 cm^−1^, which present significant differences from a preliminary visual spectral investigation (Fig. 1d). The peak positioned at 748 cm^−1^ is mainly related to the O–O stretching in oxygenated proteins and glycoproteins including mucines and proline rich glycoprotein and to the symmetric breathing of tryptophan (Trp)^27^, while the peak at 922 cm^−1^ is related to simple and branched carbohydrates including glucose and glycogen ^28^. Probably, the peaks of greatest interest are located at 1048 and 1126 cm^−1^ that can be respectively attributed to the to the Trp and phenylalanine signal and to the C–N and C–C stretching^29,30^. The potential attribution of other peaks and bands are listed in Table 1. The principal differences between the analysed groups in terms of intensities are indicated by the subtraction spectra performed between the averages Raman signals obtained (Fig. 2). The highest differences can be observed in the comparison between the CTRL group with the COV+  and COV− groups (Fig. 2a,b). All the differences in intensities (∆I) were considered for ± 0.005 ∆I, identifying peaks at 748, 897, 922, 1048, 1126, 1249, 1317 and 1348 cm^−1^. Interestingly, peaks at 1048 and 1126 cm^−1^ dominate the subtraction spectra, with important differences between the CTRL experimental group and the COV+  and COV− respectively (Fig. 2 a,b). The strong signal in correspondence of these two regions is normally associated with an environment rich in aromatic amino acids, in particular tryptophan and phenylalanine (1048 cm^−1^, Table 1). Interestingly, the same two peaks were also identified as characteristic signals from viruses of the coronavirus family, probably being involved in the viral protein structure or with the interactions with physiologically expressed molecules^31^. Recent studies conducted on SARS-CoV-2 and on other types of coronaviruses demonstrated the important presence of aromatic amino acids, including Trp, in the virus spike glycoproteins, identifying the so-called Trp-rich regions involved in the interaction between the virus and the receptor Angiotensin Converting Enzyme type 2 (ACE2)^32–34^. In terms of Raman signal, an abundance of aromatic amino acids and saccharides in saliva could explain the different intensities revealed in the relative regions for COV+  and COV− experimental groups (Fig. 2a,b). The presence in COV+  saliva of potential viral particles in large amount can be easily explained by the high viral title reached during the COVID-19 infection with the oral cavity as one of the first infection sites, together with the upper and lower respiratory tracts due to the high expression of ACE2 receptor^4,35^. Moreover, the attribution of the principal differences to protein species and modifications could be explained also with the expression of different molecules of the immune response in the first and late stages of the infection. The complex and specific expression pattern of immunity proteins, such as IgA, IgM and IgG, can last up to 3 months leading to the detectable signals both in serum and in saliva^10^. Specifically, saliva represents a reliable biofluid for the detection being involved in the first phases of infection with the persistent presence of biological molecules directly involved in the SARS-CoV-2 presence^36,37^. Clinical data collected from COV- patients, revealed periods of negativization between 8 and 90 days explaining the persistence in the Raman signals of differences when compared with CTRL and COV+  (Fig. 2b,c).

**Table 1:** Clinical characteristics of the subjects involved in the study.

	COV+	COV−	CTRL
Number	30	38	33
Age (p = 0.174)	70.8 (± 13)	71.3 (± 14.4)	61.9 (± 13.3)
Male Gender (p = 0.078)	15 (50%)	18 (47.3%)	22 (66.6%)
Severity (CIRS)	1.8 (± 0.55)	1.6 (± 0.51)	–
Neg. time (days)	–	40.16 (± 18.9)	–
Comorbidities (CIRS)	4.3 (± 2.7)	2.8 (± 1.9)	–
**Severity (Chen et al.)**			
*Stage*			
0	3 (10%)	–	–
1	7 (23%)	–	–
2	9 (30%)	–	–
3	7 (23%)	–	–
4	4 (13%)	–	–

Data are presented as average with the standard deviation (± SD) or percentage (n %) and with the two sided p-values (p). Differences between the groups were analysed using Mann–Whitney test or One-Way ANOVA test for continuous variables and Chi-square test or Fisher Exact test for categorical variables. Severities and comorbidities were considered as described by Chen et al. ^46^ and following the CIRS classification. Neg. Time represents the time between the first positive nasopharyngeal SARS-CoV 2 test and the last negative test.

These results are confirmed by the less defined differences in terms of peak intensities in the subtraction spectrum of COV+  and COV− regarding the peaks at 1048 and 1126 cm^−1^ (Fig. 2c). In this case, it is possible to observe a different trend of the subtraction spectrum, with bands related to lipids (509 cm^−1^), carbohydrates (922 and 1155 cm^−1^) and structured proteins (1317 cm^−1^) tending to the COV+  groups respect to the COV-. With the information acquired from the subtraction spectra, it is possible to identify four regions of particular interest in the spectra acquired from CTRL, COV+  and COV− groups (Fig. 2d), where the trends of the curves are modified by differences in intensities and in presence of specific peaks.

### Multivariate analysis and parameters extraction

**Figure 3:**
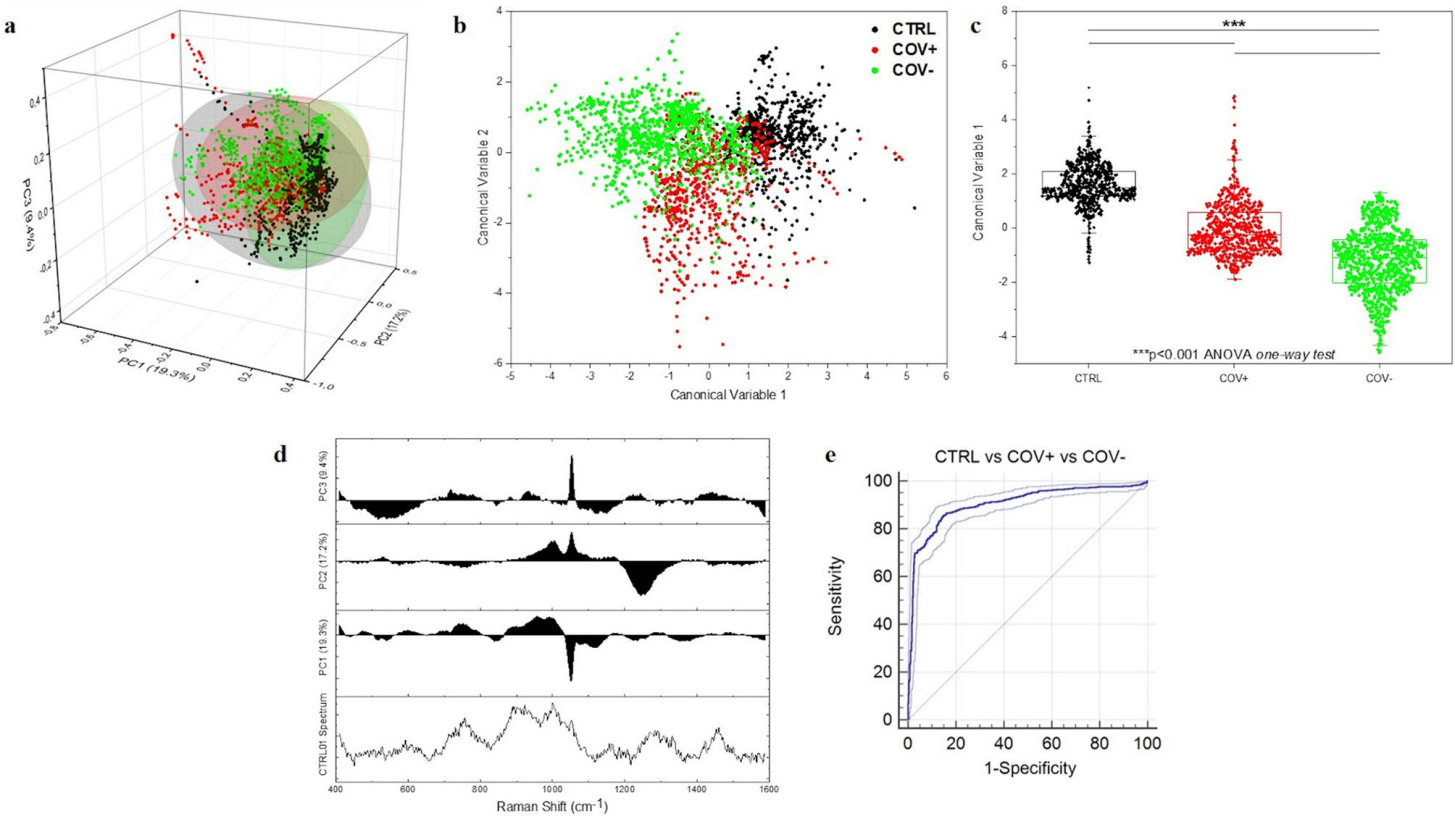
**(a)** Results of the Principal Component Analysis with the dispersion of the first three PCs values with the relative 95% confidence interval ellipses for the three experimental groups and **(b)** linear discriminant analysis (LDA) representing the spatial distribution of the two CVs extracted with the group means. c) Statistical analysis of the CVs extracted from CTRL (n = 33), COV+  (n = 30) and COV− (n = 38). *** p-value < 0.001, One-way ANOVA test. **(d)** Graphical representation of the PCs loadings respect to one of the CTRL spectrum and **(e)** receiver operator characteristic (ROC) curve with the relative 95% confidence interval of the classification model CTRL vs COV+  vs COV−. Area under the curve = 0.9; Standard error = 0.008; Significant level p < 0.001 for area = 0.5 calculated following Delong et al. ^41^.

In order to verify if the differences observed are able to discriminate the experimental groups in a classification model, we performed a Multivariate Analysis (MVA) to reduce the spectral data dimensionality and to train a Leave-One-Out Cross-Validation (LOOCV) model. The results of the Principal Component Analysis (PCA) and Linear Discriminant Analysis (LDA) on the Raman database are presented in Fig. 3. The decision to extract and represent only the first three Principal Components (PCs) relies on the cumulative loading obtained (45.9%), with significant loading attributed to the peak at 1048 cm^−1^ (Fig. 3a,d). The PCs 3D distribution shows a partial data overlapping, with distinguishable clustered regions (Fig. 3a). The CTRL group revealed a more dispersive data distribution respect to the COV+  and COV−, more grouped and centred at defined positions, probably due to the common expression of biological molecules related to the immune system in the early and late phases of infection ^10^. The LDA method was used considering the first 15 PCs as input to LDA (PCA-LDA approach), in order to maximize the variance between the data and building at the same time a classification model based on the LOOCV of the Canonical Variables (CVs). Figure 3b shows the results of the LDA, representing the spatial dispersion of the two extracted CVs. Differently from the results obtained in PCA, in this case, the CVs extracted from CTRL, COV+  and COV− show a distinct distribution with the group means highly separated in terms of relative distance (Fig. 3b). Interestingly, the CTRL CVs present the most grouped distribution respect to the other two groups, leading to the fast discrimination of the data points. The statistical analysis performed on the CVs revealed a significant difference (p < 0.001) between each involved group, demonstrating the capability of RS to discriminate the signal collected from saliva of patients affected by COVID-19 from healthy subjects and obtaining information about the past infection (Fig. 3c). The potential application of the presented technique could be of crucial importance for the discrimination of current or past infection, accompanying the serological tests used nowadays.

One probable explanation of the statistically different data distribution could be found in the physio/pathological modifications of circulating molecules when the subject is in contact with the virus. These modifications regard principally proteins, lipids, glycoproteins and their linked carbohydrates which can represent the circulating structural part of the virus, assembled and functional (COV+  group) or fragmented and non-functional (COV− group), but still circulating during the convalescence and weeks after the infections (Fig. 2 and Table 1) ^42–44^. Another potential explanation, which does not exclude the previous, can be found in alterations in the host’s proteins and lipids expression during the COVID-19 onset. The immunological response during and after the infection, induces numerous lasting changes in the expression patterns of immunological circulating cells, specific antibody presence and levels, cytokines and inflammatory mediators. These molecules, in particular cytokines and inflammatory mediators, together with the virus replication and penetration into tissue cells, induce the cell stress responses altering the redox balance and consecutively inducing damages due to Radical Oxygen Species (ROS) on lipids ^45^. All these scenarios could explain the altered salivary distribution pattern between the analysed experimental groups, determined by the high sensitivity of RS. The global results of the MVA provided variables, PCs and CVs, which can be uniquely attributed to the relative spectrum of origin, identifying in this way a set of parameters that can be used for the characterization of a classification model.

### Single spectra classification model

The result of the LDA is a set of variables that best separates the categories imposed in the supervised discriminant analysis. In order to understand the different parameters involved in the discrimination of the three experimental groups (CTRL, COV+  and COV−), we performed the LOOCV on four different scenarios. The first three analysis were performed on the binary discrimination of CTRL vs COV+  , CTRL vs COV− and COV+  vs COV−, while the conclusive discrimination was performed on the ternary model between CTRL, COV+  and COV−. The results in terms of accuracy, precision, sensitivity, specificity, Matthews Correlation Coefficient (MCC) and Error-Rate for cross-validation sum on the single spectrum (ER) are reported in Table 2.

**Table 2:** Attribution of the most prominent signals revealed from the Raman salivary analysis (± 5 cm^−1^).

Position of the Raman peak	Attribution
509 cm^−1^	Phosphorylated protein and lipids
577 cm^−1^	Trp, Cys
716 cm^−1^	Phospholipids
748 cm^−1^	O–O stretching, symmetric breathing of Trp
897 cm^−1^	Mono and disaccharides C–O–C skeletal modes
922 cm^−1^	Glucose/glycogen
1000 cm^−1^	Phe ring modes
1048 cm^−1^	C–N and C–C protein stretching
1126 cm^−1^	Trp, Phe
1155 cm^−1^	Glycogen
1249 cm^−1^	Secondary bands of amide III
1288 cm^−1^	Phosphodiester groups in nucleic acids
1317 cm^−1^	Amide III (α-helix structures)
1384 cm^−1^	C–H rocking in lipids
1453 cm^−1^	General fatty acids, C–H stretching of glycoproteins

Based ^26,28,30,38–40^.

*Trp* tryptophan, *Cys* cysteine, *Phe* phenylalanine.

The classification between the CTRL group respect to the COV+  and COV− groups presents the highest values in terms of discriminatory power, with values of accuracy, precision, sensitivity and specificity always over the 95% and with the lower associated error rate for the attribution of the single spectrum (2.2%). The result is confirmed also by the MCC values, always close to one (Table 2, CTRL vs COV+  and CTRL vs COV−). A slight decrease in the classification model performances can be observed in the discrimination between COV+  and COV− with values between 90 and 95% for accuracy, precision and specificity and 86.9% for the sensitivity (Table 2, COV+  vs COV−). In this case, the classification model loses sensitivity in the measure of positive subjects correctly identified as COV+  , probably due to the partial overlap in the CVs data distribution used as base for the model (Fig. 3b). The situation is confirmed by the MCC and ER indexes, which are respectively lower and higher compared to the previous discrimination models. The partial similarity between COV+  and COV− is reflected by the slightly higher difficulty of the model in the last ternary discrimination, with values of accuracy, precision, sensitivity and specificity of respectively 87.6%, 83.6%, 83.7% and 92.2% (Table 2, CTRL vs COV+  vs COV−). The situation is proved by the lower MCC (0.79) and the higher associated ER (15.4%). The higher specificity rate indicates that the system is able to discriminate the fraction of negatives correctly attributed to the negative groups (CTRL and COV−), but it presents some attribution problems regarding the identification of the positives including a higher number of spectra in the COV+  group respect to the true value. The 15.4% in ER for the ternary model is an average error-rate sum associated for the entire binary classification model, with the addition of the uncertainty in the partial attribution in CTRL group. The created model presents a high certainty of true negative spectra attribution (CTRL and COV−), while presents more uncertainty for the ultimate attribution of the true positive samples (COV +). Figure 3e shows the Receiver Operator Characteristic (ROC) curve of the created model CTRL vs COV+  vs COV−. The area under the curve was 0.9 (p < 0.001) demonstrating the potential application of RS in the discrimination of subjects affected by COVID-19. The single spectra approach proposed for the classification model is useful to understand the discriminatory power of the collected Raman database and to obtain results about the differences in Raman signatures of different experimental groups.

### Correlation of the Raman data with clinical parameters

**Figure 4:**
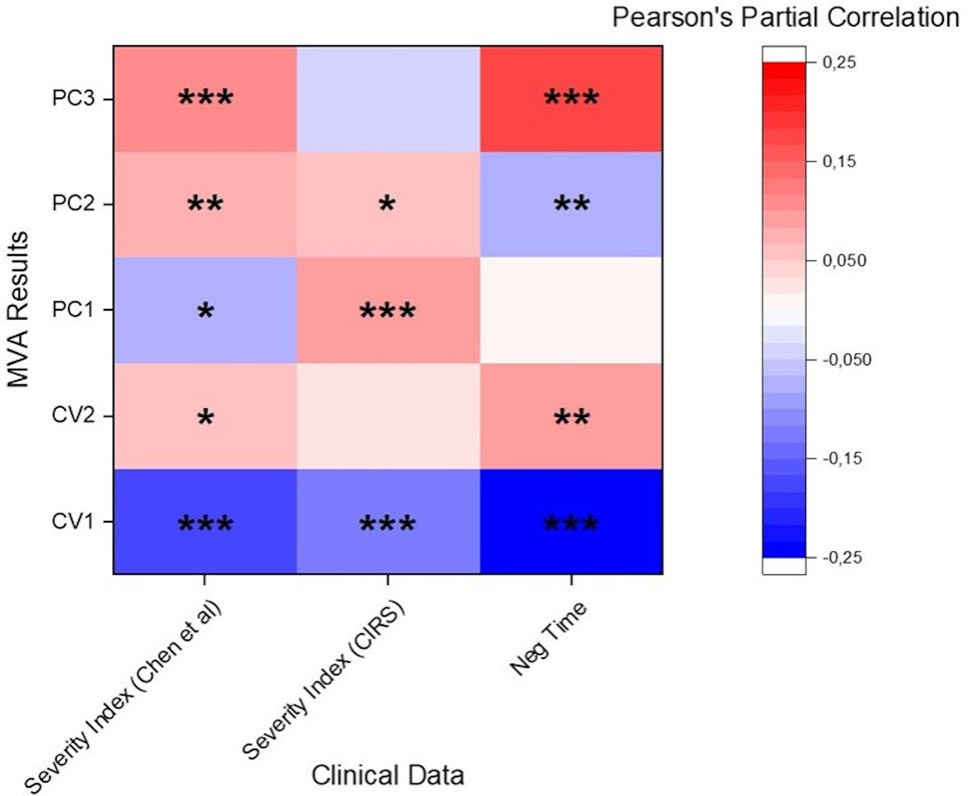
Heat map representing the partial correlation (Pearson’s coefficients) with the relative significance of Canonical Variable 1 and 2 (CV1, CV2) and Principal Components 1, 2 and 3 (PC1, PC2, PC3) correlated with the Chen et al. ^46^ and CIRS severity indexes and with the time between the first positive SARS-CoV-2 assay the last negative (Neg Time, only for COV+  group). Age, sex and the CIRS comorbidity scale were used as control covariates for the partial correlation. * p < 0.05, ** p < 0.01 and *** p < 0.001, Pearson’s test.

In order to evaluate and to assess the reliability of the proposed methodology, the data extracted from the Raman database through MVA were correlated with the severity indexes of Chen et al.^46^ and Cumulative Illness Rating Scales (CIRS) and, only for the COV+  patients, with the time between the first positive SARS-CoV-2 assay and the last negative one (Fig. 4). The correlation step was carried out using as control covariates age, sex and CIRS comorbidity index of the patients, removing possible relationships due to the demographic or physio/pathological state not related to the COVID-19. Interestingly, a great part of the MVA data correlate with the clinical parameters, with CV1 that demonstrated the strongest statistical correlation with the scales related to COVID-19 severity (Chen severity p-value = 1.4 × 10^–11^; CIRS severity = 1.2 × 10^–6^) and the infection period (p-value = 9.1 × 10^–12^). In the same way, PC3 demonstrated strong correlation with the Chen severity index and with the negativization time, but not with the CIRS severity scale. The PCs represent independent directions, with their own specific loading on the result (Fig. 3d), applied to maximize the variance between the data dispersions. Their correlation with different clinical parameters indicate a strong dependency of the Raman data, and consecutively of the variables extracted after the MVA, with the physio/pathological state of the patients confirming the reliability of the Raman methodology applied for the saliva analysis.

### Patient classification model

**Figure 5:**
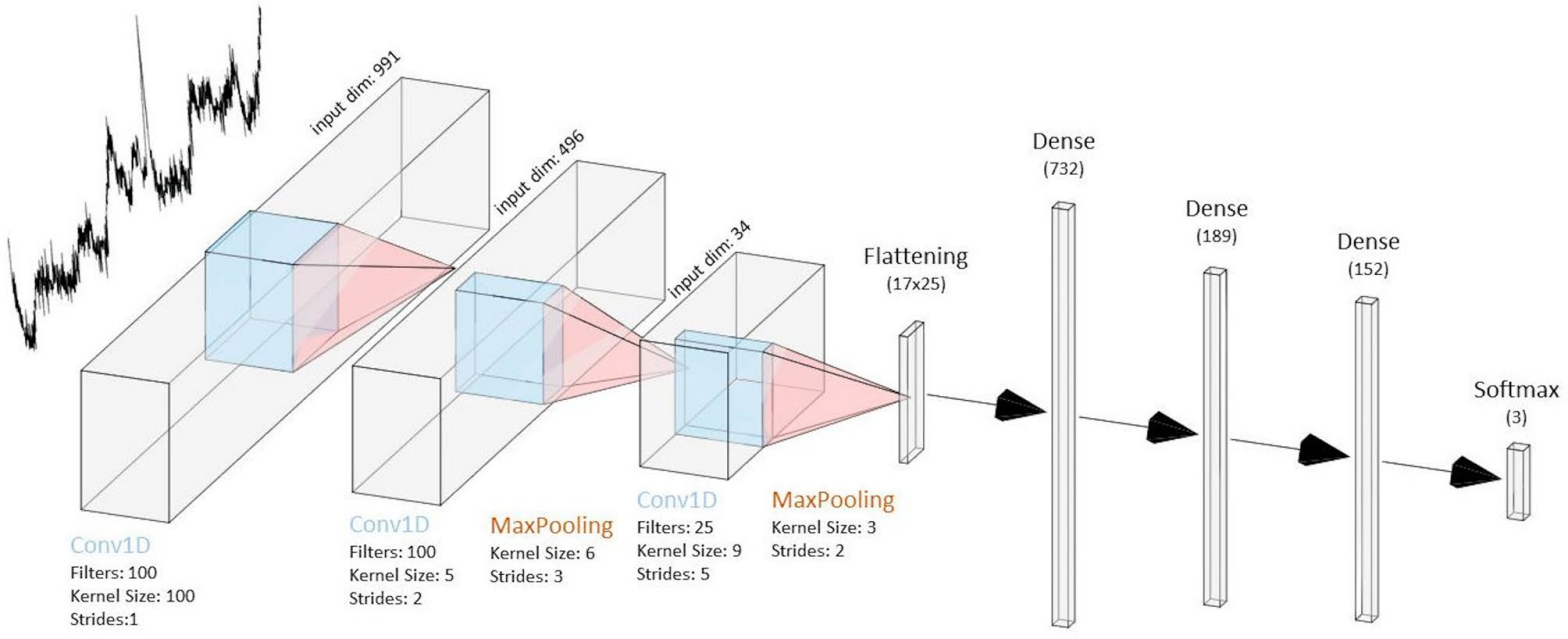
Schematic representation of the 1D-CNN model configuration obtained through the hyper parameters optimization process.

Up to now, we’ve been using the PCA-LDA approach to classify the single Raman spectra. The PCA-LDA-based classification model is suitable for the preliminary investigation of the data robustness, identifying through the LOOCV and data correlation, if the created Raman dataset can be adopted for the creation of a single spectrum classification model. Nevertheless, the final goal of a diagnostic application is the patient-level classification. In practice, all the Raman spectra pattern belonging to a certain patient may only be included either in the training or in the testing set, to avoid overfitting problems. To this extent, we applied Leave-One-Patient-Out Cross-Validation (LOPOCV), which is the patient-level version of LOOCV, where the Raman spectra belonging to all patients but one are included in the training set. As expected, by applying this evaluation method at a patient-level, the performances of classification models substantially decrease. In order to maintain good performances more sophisticated ML and DL models, such as Support Vector Machine (SVM), Random Forest (RF), Extreme Gradient Boosting (XGB), and Convolutional Neural Networks (CNNs) have been implemented and evaluated. CNNs revealed to be the most promising approach and the proposed CNN architecture with the fine-tuned hyper parameters was selected through Sequential Model Based Optimization (SMBO). The resulting model consisted of three 1-D convolutional layers for the feature extraction and three fully connected layers for the classification, with the final configuration represented in Fig. 5. It is important to notice that, while the ML baseline models were trained on normalized pre-processed data, the DL model was tested on both raw (without the pre-processing pipeline) and processed spectra. According to the data reported by Liu et al. ^47^, the CNNs were able to reach competitive performances also with the raw data, despite the low-volume high-dimensional dataset.

Consequently, the classification task was performed avoiding several time-consuming data processing steps and limiting the computational time for the results. The CNNs architecture has been trained on the 3-classes classification problem, producing in a probability distribution related to the experimental group attribution, with the highest probability taken as prediction of the corresponding label. The scores of performances reported in Table 3 are evaluated using Leave-One-Patient-Out Cross-Validation (LOPOCV), in contrast to the regular LOOCV applied in many studies ^48^ and in our preliminary analysis with the PCA-LDA model reported in Table 2. This enables diagnostic applications, associating the Raman spectrum not only to the pathology but also to the patient.

**Table 3:** Accuracy, precision, sensitivity, specificity, Matthews correlation coefficient (MCC) and error-rate for cross-validation sum on the single spectrum (ER) of the four classification models created using the leave-one out cross-validation.

	Accuracy (%)	Precision (%)	Sensitivity (%)	Specificity (%)	MCC	ER(%)
CTRL vs COV+	97.8	97.8	97.5	98	0.95	2.2
CTRL vs COV−	96.8	98.8	95.5	98.5	0.93	3.1
COV+ vs COV−	91.3	92.8	86.9	95.2	0.82	8.6
CTRL vs COV+ vs COV−	87.6	83.6	83.7	92.2	0.79	15.4

Since numerous spectral samples are acquired from the same individual, the labelling decision for a single patient can be taken based on its entire spectral set. As result, the discriminatory power in terms of accuracy, sensitivity and specificity were of respectively 89%, 89% and 95% (Table 3, patient-level). We noted that, among the misclassified patients, four were affected by severe comorbidities (but still matching the inclusion criteria). By excluding these patients, the DL model accuracy increased to 92%, with sensitivity and specificity of 91% and 96%, respectively (Table 3, patient-level COV+  = 26). In both cases, DL models outperform the classical ML algorithms, which only obtained up to 86% accuracy. Observing the results obtained from the multi-class and binary classifications regarding the attribution of COV+  , it is clear that the misclassified COVID-19 patients are generally classified in the wrong way as COV− subjects, while CTRL and COV− can be well discriminated (Table 3). These results agree with the findings exposed after the application of the MVA on the single spectra, where a higher error rate on the data attribution to the experimental groups were higher respect to the related binary classification models (Table 2). These results clearly indicate similarities in the COV+  and COV− spectra respect to the CTRL group, with signal trends that make more difficult the discrimination and the attribution to the correct group.

## Discussion

In this work, an alternative Raman approach was presented for the discrimination of current or past infection by SARS-CoV-2 from a really simply and safely collectable biofluid such as saliva. The sample processing and analytical procedure is simple, automatable, and highly informative and can be performed obtaining results in few minutes. The discrimination ability of the RS relies in the wide range of information that can be obtained from a single analysis, with the lowest concentration detectable up to 10^–15^ M in specific SERS regimen, in which complex SERS substrates have been applied^49^. With the SERS approach, the “global” information regarding the analysed biofluid can be collected, obtaining a whole signal from all the biochemical species present on the base of their concentration, environment, chemical nature, modifications, mutations, alterations and interactions. In addition, slight changes in the expression pattern of these molecules could provide a deformation of the normal trend in curve shape, which can be detected, analysed, computed and used to build a classification model. The numerous databases available in literature on the Raman analysis of biofluid can be questioned to explain the various peaks and bands present in the single spectrum, and to understand the potential molecular differences encountered between, for example, the pathological state versus the physiological state of the selected experimental groups. With the rapid and continuous increase of confirmed and severe cases of COVID-19, timely identification of infected subjects might be helpful using a fast and minimal invasive procedure such as RS on saliva. In the same way, the identification of subjects with a past infection (with or without symptoms) could be fundamental to create epidemiological maps useful for the infection confinement. The proposed procedure provides information regarding the presence, concentration and modification of molecules of interest including nucleic acids and proteins, all with a single fast analysis of saliva. Moreover, the easy procedure, the robust and reproducible analytical protocol and the cheapness of the Raman substrate depict a potential ideal method for the discrimination of COVID-19 onset. Thus, overcoming the technical limitations present in the methodologies used nowadays for the detection of viral nucleic acids (nasopharyngeal SARS-CoV-2 test) and antigens (serological capillary assay), or for the identification of specific circulating IgM and IgG (serological ELISA). The high cost of the bench Raman instrument might be attenuated implementing the system with a portable version, widely available commercially with spectral resolutions and Raman shift ranges comparable with the bench version. The application of a portable Raman combined with the implementation of the proposed classification model with a larger cohort of COVID-19 patients, could lead to the creation of a point of care able to assess the positivity to the SARS-CoV-2 infection and to predict the severity of the disease in terms of respiratory clinical manifestations on the base of the detected stage. Once assessed the discrimination capability of our models, the performances are expected to drastically increase together with the input of new Raman and clinical data of more patients. In fact, introducing more data about the Raman signal collected from saliva of different patients belonging to specific experimental groups (e.g. longitudinal clinical course, comorbidities or asymptomatic), the classification model will be potentially able to discriminate more accurately the infection onset and also to predict the severity stage of the respiratory tract. The optimal scenario includes different Raman workstations connected through an online learning network, able to analyse and to furnish a huge amount of information for the system training. The correlations with the data extracted from the Raman database through MVA are of crucial importance for two reasons: the first one is an indication of the reliability of the proposed methodology, which explains the intrinsic relationship between the Raman analysis and the complex biochemical composition of saliva under pathological conditions. The second reason relies in the possibility to evaluate the COVID-19 severity or the negativization time on the base of a simple salivary analysis. All these information were adopted to train two classification models, the first one was evaluated for the characterization of the specific Raman fingerprint of COV+  and COV− patients (MVA, single spectrum classification model, Table 2) while the second one was intended for the definition of the subject condition (ML/DL, patient classification model, Table 3). Both the models highlighted a high accuracy rate in the attribution of the data in the exact experimental group, with variations in the discrimination of single categories. CTRL and COV− were attributed with higher discrimination power respect to the COV+  that presented slightly lower percentages of accuracy, sensitivity and specificity. The decrease was due to the attribution of misclassified patients to the COV− class, indicating a close relationship between the two groups. The slight increase in the discrimination power observed removing from the training the four patients with relevant comorbidities, reveals the reliability of the applied methodology that is able to attribute the data to the right experimental classes also in complex clinical pictures. The main advantage of the combined RS and DL approach relies in the increase in discriminatory power with the enlargement of the data provided in terms of higher patients’ number and new experimental classes. Probably, also the lower number of COV+  patients plays a crucial role in this attribution and, for this reason, the recruitment of a higher number of COVID-19 infected subjects is in progress. In conclusion, our results demonstrated the existence of a characteristic salivary COVID-19 Raman fingerprint, which can be used to train and test a classification model able to discriminate between CTRL and COV−. The deep investigation of the collected data revealed partial similarities of the salivary Raman spectra patients affected by COVID-19 and subjects with a history of infection but current negative SARS-CoV-2 assay, confirming the persistence of molecules related to the SARS-CoV-2 presence also months after the infection^10,50–52^. The proposed methodology has the potential to quickly and accurately diagnose the COVID-19 onset, reflecting the time and the severity of the infection, analysing an easily and safely collectable biofluid such as saliva.

## Methods

### Patients and clinical data

The study was approved and conducted following the directives of the Ethical Committee institutional review board at IRCCS Fondazione Don Carlo Gnocchi on 16th April 2020, which required the application of the criteria of the Helsinki declaration and of all the relevant regulations regarding the enrolment of human patients. None of the patients was presented in prior publications regarding their clinical characteristics. Saliva samples, health records and clinical data were acquired at IRCCS Fondazione Don Carlo gnocchi ONLUS, Milan, Italy and Centro Spalenza Hospital, Rovato, Italy between 16th April 2020 to July 2020. The detailed description of the inclusion and exclusion criteria for patients’ selection is reported on ClinicalTrials.gov (ID: NCT04583306, The salivary Raman COVID-19 fingerprint). The COVID-19 diagnosis was conducted following the WHO guidelines, declaring a positive case after the positive result of sequencing or rRT-PCR assay of SARS-CoV-2 for nasopharyngeal swabs. The patients were considered COVID-19 negativized after two consecutive tests with negative results, recording the time of negativization as the time between the last SARS-CoV-2 positive test and first negativization assessment (two consecutive negative SARS-CoV-2 tests). Demographic data of patients and healthy subjects were recorded, as well as eventual comorbidities (e.g. diabetes, cardiovascular diseases, arterial hypertension, neurological disorders, respiratory obstructive diseases and cancer or concomitant viral or bacterial infections) and pharmacological and/or invasive or non-invasive respiratory therapies. Patients clinical classification and complication definitions were carried out following the guidelines presented by Chen et al.^46^*.* Briefly, patients were defined as asymptomatic (0), mild (1), moderate (2), severe (3) and critical (4) cases on the base of the observed clinical picture.

The CIRS for severity and comorbidities were calculated. Age and sex correlated healthy subjects were recruited. The total number of subjects involved in the study was 30 patients affected by COVID-19 (COV+  ; n = 30), 38 subjects negative to the SARS-CoV-2 test with an ascertained episode of COVID-19 (COV−; n = 38) and 33 age and sex correlated CTRL (n = 33). All participants provided written informed consent. Table 4 reports the main characteristics of the subjects involved in the study. All methods and procedures reported in this study were carried out in accordance with relevant guidelines and regulations.

**Table 4:** (Ternary) CNN accuracy, sensitivity and specificity at the patient level of the ternary (COV+  vs COV− vs CTRL) classification model using leave-one patient out cross-validation, with the related COV+  sensitivity and specificity percentages. (Binary) CNNs accuracy, sensitivity and specificity at the patient level for the binary (COV+  vs CTRL, COV+  vs COV− and COV− vs CTRL) classification model.

	Accuracy (%)	Sensitivity (%)	Specificity (%)	COV+ sensitivity (%)	COV+ specificity
**Ternary**					
Patient-level	89	89	95	83.3	96
Patient-level (COV+ = 26)	92	91	96	85	96
**Binary**					
COV+ vs CTRL	89	83	94	–	–
COV+ vs COV−	89.6	83	93	–	–
COV− vs CTRL	91.5	89	94	–	–

### Sample collection

Saliva collection was carried out using Salivette swabs (Sarstedt, Germany), following manufacturer’s instructions. Briefly, the swab was placed in the subject’s mouth and chewed for 60 s to stimulate salivation. The cotton swab was then closed into the provided plastic tube and centrifuged for 2 min at 1000*g* in order to remove eventual food debris and to collect saliva. Saliva samples were stored at − 20 °C. To limit the variability in biomolecules concentration in saliva, the collection procedure was performed at fixed time-points after breeding and teeth brushing. Pre-analytical parameters, including collection time, time between the collection of saliva and the Raman analysis and storage temperature, were recorded. All the data were anonymized preserving the subject privacy. A saliva drop (3 µl) was deposited on a glass slide covered with commercially available aluminum foil, in order to achieve the SERS effect, and dried at room temperature for 10–15 min. Aluminum foil was purchased from Merck KGaA (Germany) and used as received to cover the glass substrate for the SERS analysis.

### Raman analysis

SERS spectra were acquired using an Aramis Raman microscope (Horiba Jobin Yvon, France) equipped with a laser source of 785 nm with laser power fixed at 512 mW. The salivary Raman measurements were performed following a slightly modified protocol developed by Carlomagno et al.^15^. Briefly, before each analysis, the instrument was calibrated using the reference band of silicon at 520.7 cm^−1^. SERS spectra were collected following a 25-points rectangular map (60 µm × 40 µm) for each saliva sample, positioned between the centre and the edge of the dried saliva drop using a 50 × objective (Olympus, Japan). Spectra were acquired in the region between 400 and 1600 cm^−1^ with a spectral resolution of 0.8 cm^−1^. Other parameters were predisposed accordingly to the followed protocol^15^. The incorporated software LabSpec6 (version 6.4.3.35, https://www.horiba.com, Horiba Jobin Yvon, France) was used for the map definition and spectra acquisition.

### Single spectrum classification model

All the acquired spectra were fit with a fourth-degree polynomial baseline, considering 79 points at the lower spectral level, and normalized by unit vector using the software LabSpec6. The aluminum substrate contribution was removed from each signal, applying at the same time a second-degree Savitzky-Golay smoothing in order to remove the artefact spikes. All the spectra were resized, avoiding large peak shifts due to external factors (e.g. thermal shifts). Artefact spectra, including fluorescence-dominated or saturated signals, were manually removed from the dataset. At least 20 spectra were preserved for each salivary sample. The specific Raman fingerprint was calculated performing the average spectrum of each experimental group and comparing the resultant spectra through subtraction with error dispersion, individuating the significant intensities variations at the ± 0.005 ∆I level (Fig. 2a–c)^53^. The single spectra classification model was created and trained using the MVA approach, consisting in consecutive PCA and LDA on the created Raman database, using the whole collected spectra. The PCA was applied in order to reduce data dimensionality and to highlight and define relevant dispersion trends. The first three PCs were extrapolated and used for the data representation, explaining the cumulative loading of PCs of 45.9%. The first 15 PCs were used as input to the LDA process, obtaining two CVs for the three experimental groups ^54^. LDA was used to discriminate the data and to maximize the variance between the groups. The classification model was built through the LOOCV approach, avoiding the over or under fitting due to an inappropriate selection of components considered and determining the prediction of error-rate associated to the classification model ^48^. The confusion matrix test on the LOOCV results was used to determine the accuracy, precision, sensitivity and specificity of the system to discriminate the single spectrum acquired. MCC was calculated to evaluate the quality of the classification ^55^. The ROC curve was calculated using the method reported by Delong et al. ^41^, reporting the curve standard deviation with a 95% confidence interval and p-values < 0.001. Chi-square, Fisher exact test, ANOVA and Mann–Whitney tests were used to assess the differences between the experimental groups (two-sided p-value less than 0.05 were considered as statistically significant). Correlation and partial correlation analysis were performed using Pearson’s test and, in the case of partial correlation, using as correction covariates the CIRS comorbidity scales and demographic data including age and sex. The described procedures and the images were performed using Origin Pro 2018 (version b9.5.1.195, https://www.OriginLab.com, OriginLab Corp. USA) and MedCalc (version 14.8.1, https://www.medcalc.org, Medcalc Software, Belgium).

### Patient classification model

The patient classification model was created through the definition and refinement of automatic spectral pre-processing, data augmentation, application and evaluation of different ML and DL models, hyper parameters optimization and the final evaluation of the optimized selected model. Briefly, all the spectra included in the Raman database were treated as follows: removal of the outliers; realignment on the peak at 1001 cm^−1^; resampling of each spectrum on a grid of 900 points between 400 and 1600 cm^−1^; subtraction of the background signal of aluminum (band centred at 1250 cm^−1^); removal of signals collected from cosmic rays applying the Whitaker–Hayes algorithm^48,56,57^. Concluding the pre-processing pipeline, all the spectra were normalized evaluating four different techniques including Standard Normal Variate, Min–Max, Max value and L2. The results for these methods demonstrated no significant differences between the processes. Data augmentation was applied in order to generate new synthetic spectra applying variations and distortions to the original data by injecting a small contribution of Gaussian noise to the original spectrum at random wavenumbers. As consequence, we were able to improve the performances of the classification model, overcoming the scarcity of the data^58^. It is worth noticing that data augmentation has been applied only to the training set, not to the test set. Further details for the data augmentation mechanism and its training application are reported in Appendix A. For the implementation of ML and DL models, different techniques were evaluated including SVM, RF and XGB. Before the application of the ML-based techniques, both data pre-processing and data reduction steps (e.g. PCA) are needed, while the DL methods are able to directly handle unprocessed data, making the development of the model faster and more reliable. SVM, RF and XGB were implemented as baseline and compared with DL models, in particular with CNNs. The hyper parameters optimization was performed in two different phases: DL structure optimization, selecting the number of convolutional or dense layers, and fine-tuning of their hyper parameters. The two phases were applied using SMBO with Gaussian processes and RF regressions as base estimators, all in combination with lower confidential bound acquisition functions. All the optimizations were performed in 10-folds cross-validation and the target function was minimized on the average classification error. The CNN model is trained for a maximum of 200 epochs using early stopping. Adam optimizer and batch size 128 are adopted. The performances of the classification model were evaluated considering their ability in the CTRL, COV+  and COV− discrimination. Each subject’s label was determined according to the majority class assigned to her/his spectra. Therefore, we built a single spectra ML/DL classifier whose predictions were then aggregated at patient level. In order to overcome any classification bias due to the eventual arbitrary test-set choice, we applied the LOOCV. In this way, the classification model went through a robust and stable procedure where each test-fold is composed of the entire set of spectra from a single patient that guarantees a most accurate estimation of the model performances. For the classification model operating at the patient level, the data collected from four COV+  patients were evaluated, trying to include or exclude them from the training. Both the results are reported. All the procedures and methods were coded using Python (version 3.9.1, https://www.phyton.org) and implemented through Scikit-Learn Library and Keras, the Tensorflow high-level API ^59,60^.

## Supplementary Information

Supplementary Information.

## Data Availability

The data supporting the main findings of this study are available from the corresponding authors upon reasonable request. The analytical protocol is available on ClinicalTrials.gov, ID: NCT04583306, The salivary Raman COVID-19 fingerprint.

## Code availability

The codes and algorithms used in this work are available after reasonable request from V.M. and D.B.
