# Supplementary material for: COVID-19 salivary Raman fingerprint: innovative approach for the detection of current and past SARS-CoV-2 infections
Source: Sci Rep. 2021 Mar 2;11:4943. doi: 10.1038/s41598-021-84565-3 (PMC7925543; doi:10.1038/s41598-021-84565-3)
Supplement: Supplementary file 1 — Supplementary Information. [file 41598_2021_84565_MOESM1_ESM.docx]

**Supplementary Information**

**COVID-19 salivary Raman fingerprint: innovative approach for the detection of current and past SARS-CoV-2 infections**

*C. Carlomagno ^1^, D. Bertazioli ^2^, A. Gualerzi ^1^, S. Picciolini ^1^, P. I. Banfi ^1^, A. Lax ^1^, E. Messina ^2^, J. Navarro ^1^, L. Bianchi ^1^, A. Caronni ^1^, F. Marenco ^1^, S. Monteleone ^1^, C. Arienti ^1^, *****M. Bedoni ^1^.

^1^ IRCCS Fondazione Don Carlo Gnocchi ONLUS, Via Capecelatro 66, 20148 Milan (Italy)

^2^ Università di Milano – Bicocca, Viale Sarca 366, 20126 Milan (Italy)

*Corresponding authors: Cristiano Carlomagno (ORCID: https://orcid.org/0000-0002-5543-0505); Marzia Bedoni (ORCID: https://orcid.org/0000-0003-2618-3661)

Corresponding author email: ccarlomagno@dongnocchi.it; [mbedoni@dongnocchi.it](mailto:mbedoni@dongnocchi.it)

**Appendix A**

**Hyper-parameter optimization details and training specifications for the CNN model.**

The detailed results of the hyper parameter search are reported in Table 1 Supplementary, together with their relative search space. The CNN model is trained for a maximum of 200 epochs, using early stopping: therefore, for each fold of the LOPOCV, the training is stopped after 50 epochs of validation accuracy stagnation, to avoid overfitting. We exploited the Adam optimizer, scheduling its learning rate with a so-called “ReduceLearningRateOnPlateau” strategy, starting from a learning rate value lr = 0.001 and systematically halving it once the validation metrics did not improve for 10 consecutive epochs. To improve the robustness and the generalization capability of the data-hungry DL model, we generated new synthetic spectra by means of data augmentation. Indeed, it is possible to simulate the spectral imperfections and variations characteristics of the RS acquisition process by injecting a small contribution (equal ~0.1 σtrain, where σtrain is the intensity standard deviation of the training set) of Gaussian noise to the original spectra at random wavenumbers. In addition to direct noise injection, other augmentation components are applied, namely the stochastic modulation of the spectral offset and slope. The main effects include: our procedure slightly shifts the intensities, making the system invariant to weak translation along with the intensity axis; it reshapes the Raman peaks according to a certain multiplicative factor (1 ± 0.1 σtrain), and it alters the spectrum slope, from a local (close to a peak) or global perspective (slope was adjusted with a random multiplicative factor uniformly distributed in [0.95, 1.05]). In each augmentation step, the values of the additive/multiplicative parameters have been carefully chosen after an extensive exploration, having in mind that their variation range should be small enough to make sure that the generated spectra are still realistically similar to their original version, but, at the same time, large enough not to produce almost identical and therefore useless new examples. The data augmentation has been applied according to the optimized “data augmentation factor”, but only to the training set, never to the test set. Since the performances have been measured in a Leave-One-Patient-Out Cross Validation, the data augmentation has been performed online. Therefore, each training fold is augmented independently: in this way, we ensure no biases nor data leakage is introduced by the data augmentation procedure.

**Table 1 Supplementary. Summary of the optimized parameters for the COVID dataset for the final neural network hyper-parameters optimization phase with their relative search space and the final values obtained: int. refers to integer search space while logunif. and discrete unif. respectively to log-uniform and discrete uniform search space.**

| **Hyper-parameters** | **Search Space** | **Final Value** |
| --- | --- | --- |
| **CNN1 filters** | int. [l = 5, h = 150] | 100 |
| **CNN1 kernel size** | int. [l = 5, h = 100] | 100 |
| **CNN1 strides** | int. [l = 1, h = 5] | 1 |
| **CNN2 filters** | int. [l = 5, h = 120] | 100 |
| **CNN2 kernel size** | int. [l = 5, h = 30] | 5 |
| **CNN2 strides** | int. [l = 1, h = 5] | 2 |
| **MaxPool2 size** | int. [l = 2, h = 10] | 6 |
| **MaxPool2 strides** | int. [l = 2, h = 6] | 3 |
| **CNN3 filters** | int. [l = 5, h = 150] | 25 |
| **CNN3 kernel size** | int. [l = 5, h = 20] | 9 |
| **CNN3 strides** | int. [l = 1, h = 5] | 5 |
| **MaxPool3 size** | int. [l = 1, h = 10] | 3 |
| **MaxPool3 strides** | int. [l = 1, h = 4] | 2 |
| **Dropout rate (after flattening)** | discrete unif. [l = 0.1, h = 0.95, q =0.05] | 0.1 |
| **Dense units 1 (first dense layer)** | int. [l = 32, h = 1024] | 732 |
| **Dropout rate** | discrete unif. [l = 0.1, h = 0.95, q =0.05] | 0.3 |
| **Dense units 2** | int. [l = 32, h = 1024] | 189 |
| **Dense units 3** | int. [l = 32, h = 1024] | 152 |
| **Batch size** | binned int. [l = 2, h = 512] | 128 |
| **Augmentation factor** | int. [l = 1, h = 100] | 30 |

**Appendix B**

**ML baseline results.**

Table 2 Supplementary reports the results of our extensive testing and comparison of ML models at the patient-level LOPOCV training on the ternary classification problem (COV+ vs COV- vs CTRL).

**Table 2 Supplementary. Ternary accuracy, sensitivity, specificity at the patients ternary level (COV- vs COV+ vs CTRL)**

| **Model** | **Accuracy** | **Sensitivity** | **Specificity** |
| --- | --- | --- | --- |
| **PCA-LDA** | 80% | 79% | 90% |
| **SVM** | 78% | 78% | 89% |
| **RF** | 77% | 77% | 88% |
| **XGB** | 86% | 85% | 93% |
